# Child abuse and neglect: psychiatric and neuro-biological consequences

**DOI:** 10.1186/1824-7288-40-S1-A32

**Published:** 2014-08-11

**Authors:** Pietro Ferrara

**Affiliations:** 1 Institute of Pediatrics, Catholic University of Sacred Heart, Rome, Italy and Campus Bio-Medico University, Rome, Italy

## 

Child abuse and neglect is a common problem that is potentially damaging to long-term physical and psychological health of children. Over the past, researchers have documented this relationship and have identified two possible mechanisms that can explain the increased incidence of childhood stress and consequent adult somatic disease: the increased incidence of health harming behaviors and causes epigenetic and other changes that predispose individuals to disease through a raised non-specific inflammatory profile [1]. Abuse survivors, as well as persons who have experienced other types of childhood adversities, are more likely to participate in high-risk behaviors [2]. Possible etiologic factors in survivors' health problems include abuse-related alterations in brain functioning that can increase vulnerability to stress and decrease immune function. Adult survivors are also more likely to participate in risky behaviors that undermine health or to have cognitions and beliefs that amplify health problems [2].

Childhood abuse and early life stress may become hard-coded into the genome, creating an epigenetic memory of events that leads to impaired health at a later date [3].

Chronic early life stress results in long-term changes in HPA (hypothalamic-pituitary-adrenal) axis function and regulation typified by hypersecretion of CRH and ACTH. The initial hypersecretion of cortisol may over time lead to blunting of the cortisol response to CRH and ACTH and relative glucocorticoid resistance. A decrease in glucocorticoid levels or impaired glucocorticoid receptor function might then lead to increased stress responsiveness [2].

Childhood abuse and neglect is also associated with reduced adult hippocampal volume, particularly on the left side, and these findings support the hypothesis that exposure to early stress in humans, as in other animals, affects hippocampal subfield development [4].

Another recent study demonstrate that children who experienced two or more types of violence exposure showed significantly accelerated telomere erosion from age-5 baseline to age-10 follow-up measurement compared with children who had one type of violence exposure or who were not exposed to violence [5].

People experience and interpret physical and emotional insults in diverse ways and many contextual factors affect the phenomenology of abuse and neglect, and in Italy there are many children victims of various types of maltreatment particularly within their own families. But we must not forget the most vulnerable children who may have even more serious consequences: those who live in foster care [6] or abandoned babies [7] that are actually little-known but that equally needs of social interventions, health and human rights.

## References

[B1] Hyland ME Alkhalaf AM Whalley B Beating and insulting children as a risk for adult cancer, cardiac disease and asthma J Behav Med 2013 36 632 640 10.1007/s10865-012-9457-6 23054177

[B2] Sachs-Ericsson N Cromer K Hernandez A Kendall-Tackett K A Review of Childhood Abuse, Health, and Pain-Related Problems: The Role of Psychiatric Disorders and Current Life Stress J Trauma Dissociation 2009 10 170 188 10.1080/15299730802624585 19333847

[B3] Tietjen GE Peterlin BL Childhood abuse and migraine: epidemiology, sex differences, and potential mechanisms Headache 2011 51 869 879 10.1111/j.1526-4610.2011.01906.x 21631473 PMC3972492

[B4] Teicher MH Anderson CM Polcari A Childhood maltreatment is associated with reduced volume in the hippocampal subfields CA3, dentate gyrus, and subiculum Proc Natl Acad Sci USA 2012 109 E563 572 10.1073/pnas.1115396109 22331913 PMC3295326

[B5] Shalev I Moffitt TE Sugden K Williams B Houts RM Danese A Mill J Arseneault L Caspi A Exposure to violence during childhood is associated with telomere erosion from 5 to 10 years of age: a longitudinal study Mol Psychiatry 2013 18 576 581 10.1038/mp.2012.32 22525489 PMC3616159

[B6] Ferrara P Romani L Bottaro G Ianniello F Fabrizio GC Chiaretti A Alvaro F The physical and mental health of children in foster care Iran J Public Health 2013 42 368 373 23785675 PMC3684722

[B7] Ferrara P Gatto A Paolillo P Vena F Ianniello F Romagnoli C Abandoned newborn: neglected phenomenon? Early Hum Dev 2013 89 Suppl 4 S45 S46

